# Early Fever Resolution in Early Childhood Influenza Treated with Baloxavir Marboxil: A Retrospective Study Compared to Those with Oseltamivir

**DOI:** 10.3390/medicina59091543

**Published:** 2023-08-25

**Authors:** Keiko Nezu, Shingo Hayashida, Nobuhiko Nagano, Seiichi Udagawa, Ichiro Morioka

**Affiliations:** 1 Department of Pediatrics and Child Health, Nihon University School of Medicine, Tokyo 1738610, Japan; kawauso@love.email.ne.jp (K.N.); hayashida.shingo@nihon-u.ac.jp (S.H.); nagano.nobuhiko@nihon-u.ac.jp (N.N.); 2 Nezu Clinic, Tokyo 1740042, Japan; 3 Mathematics Section, Division of Natural Sciences, Nihon University School of Medicine, Tokyo 1738610, Japan; udagawa.seiichi@nihon-u.ac.jp

**Keywords:** cap-dependent endonuclease inhibitor, early childhood, fever, influenza virus, oseltamivir

## Abstract

*Background and Objectives*: Baloxavir marboxil is a novel cap-dependent endonuclease inhibitor prescribed for influenza treatment. Unlike neuraminidase inhibitors like oseltamivir, which impair viral release from infected host cells, baloxavir blocks influenza virus proliferation by inhibiting viral mRNA transcription. This study aimed to compare the effectiveness of baloxavir and oseltamivir for the treatment of early childhood influenza. *Materials and Methods*: Of 1410 patients diagnosed with influenza between 2015 and 2018 at a Japanese primary care outpatient clinic, 1111 pediatric patients aged 0–6 years who were treated with baloxavir (*n* = 555) or oseltamivir (*n* = 556) were enrolled retrospectively. The following clinical factors were compared between patients treated with baloxavir and oseltamivir: age, sex, time from fever onset to drug administration (<24 h or 24–48 h), time from drug administration to fever reduction, influenza type (A or B), and influenza vaccination before disease onset. The duration of the fever, which was used as an index of clinical effectiveness, was compared using the log-rank test. Clinical factors associated with fever duration were determined using multivariate logistic regression analysis. *Results*: Median age (3.0 vs. 2.5 years), influenza type A (99% vs. 47%), median duration from drug administration to fever resolution (1 day vs. 2 days), and influenza vaccination (done, 41% vs. not done, 65%) were significantly different between the baloxavir and oseltamivir groups (*p* < 0.001). The number of patients with a fever duration of one day was 553 (99.6%) in the baloxavir group and 6 (1.1%) in the oseltamivir group (*p* < 0.001). Baloxavir use was only significantly associated with fever duration in the multivariate analysis (odds ratio 50,201, *p* < 0.001). Apparent adverse effects were not observed in the baloxavir-treated group. *Conclusions*: Baloxavir treatment resulted in a shorter fever duration than oseltamivir treatment in early childhood influenza.

## 1. Introduction

Influenza is a common and significant pediatric infectious disease with fever and respiratory, digestive, or systemic symptoms (cough, nasal discharge, headache, pharyngeal pain, vomiting, diarrhea, malaise, myalgia, and joint pain) caused by the influenza virus [1]. The disease cures spontaneously, but the prognosis declines when complicated by pneumonia, rhabdomyolysis, encephalitis or encephalopathy, and myocarditis or pericarditis [1]. Many deaths occur annually worldwide [1].

In Japan, neuraminidase inhibitors that impair viral release from the infected host cells, such as oseltamivir, zanamivir, peramivir, and laninamivir, are commonly used to treat influenza. During the 2009 influenza A/H1N1 pandemic, the mortality rate was extremely low in Japanese pediatric patients (only three per million infected children) [2]. One possible reason was the early treatment with neuraminidase inhibitors [2]. Therefore, early treatment is important to reduce deaths and fatal complications caused by pediatric influenza.

In pediatric outpatient clinics, especially in early childhood, oral oseltamivir is used primarily in children over 2 weeks of age in Japan because the alternatives, zanamivir and laninamivir, are inhalants, and peramivir requires an intravenous line. Oseltamivir treatment is effective in reducing the time to symptom alleviation [3] and the rate of hospitalization and complications, such as respiratory illness, pneumonia, and otitis media, in pediatric influenza patients [4]. However, oseltamivir is known to cause adverse effects such as vomiting, abdominal pain, epistaxis, ear disorders, and conjunctivitis [3,5]. Antiviral-resistant strains have also been reported in oseltamivir-treated children [6,7]. Therefore, various options for the treatment of influenza are required for children.

Baloxavir marboxil is a novel cap-dependent endonuclease inhibitor prescribed for the treatment of influenza. Unlike neuraminidase inhibitors that impair viral release from infected host cells, baloxavir blocks influenza virus proliferation by inhibiting viral mRNA transcription [8]. In 2018, baloxavir was approved for the oral treatment of influenza in Japan [9] and is widely used in clinical practice. Post-marketing surveillance in Japan and other clinical studies, including randomized control trials and meta-analyses, have shown the efficacy and safety of baloxavir in adult and pediatric influenza [8,10,11,12,13,14,15,16]. For example, in a phase 3 clinical trial conducted during the 2016–2017 influenza season in adults and adolescents (CAPSTONE-1), baloxavir was superior to placebo in improving influenza symptoms and was superior to both oseltamivir and placebo in reducing the viral load in patients aged 12–64 years with uncomplicated influenza without severe side-effects [8]. The phase 3 clinical trial conducted in high-risk patients aged 12–89 years with conditions such as asthma or chronic lung disease, endocrine disorder, heart disease, metabolic disorder, and obesity also showed a similar result in that baloxavir shows superior efficacy to placebo and similar efficacy to oseltamivir in improving influenza symptoms and signs (CAPSTONE-2) [11]. In the phase 3 trial conducted in children aged 1–12 years, baloxavir showed similar clinical efficacy to oseltamivir (miniSTONE-2) [12]; however, in this miniSTONE-2 study, the dose was higher than the dose used for children in Japan. 

Although there have been some comparative studies on the efficacy of baloxavir and oseltamivir in pediatric and adolescent patients (<18 years of age) [17,18,19,20], no studies have been conducted in early childhood patients aged 0–6 years. This study aimed to compare the effectiveness of baloxavir and oseltamivir for the treatment of early childhood influenza, using the duration of fever as an index of clinical effectiveness.

## 2. Materials and Methods

### 2.1. Study Design and Subjects

This retrospective cohort study was conducted at a primary care outpatient clinic in Tokyo, Japan, with the approval of the Institutional Review Board of Nihon University Itabashi Hospital (RK-190910-10, date of approval: 20 September 2019). In this cohort study, 1410 patients who were diagnosed with influenza between January 2015 and December 2018 were enrolled. The diagnosis of influenza was confirmed by a rapid antigen test with immunochromatography using a nasopharyngeal swab (RapidTesta^TM^ Flu stick; Sekisui Medical, Co., Ltd., Tokyo, Japan). Patients with serious influenza complications such as acute encephalopathy or myocarditis were excluded because they were transported to another hospital for intensive care.

Of the 1410 patients, 299 were excluded because of age >6 years or insufficient clinical data. In total, 1111 patients with influenza aged 0–6 years were treated with baloxavir (*n* = 555) or oseltamivir (*n* = 556). Patients during the 2015–2018 season were treated with oseltamivir. After the clinical approval of baloxavir in Japan, patients during the 2018–2019 season were treated with either oseltamivir or baloxavir. Clinical factors were retrospectively collected from medical records, including age, sex, time from fever onset to drug administration (<24 h or 24–48 h), time from drug administration to fever resolution, influenza type (A or B), influenza vaccination before onset in this season, percentage of patients with upper respiratory tract symptoms (cough, rhinorrhea, or nasal obstruction feeling) 4–5 days after drug administration, and persistence of gastrointestinal symptoms (nausea, vomiting, or diarrhea).

### 2.2. Treatments and Definition

All patients received either baloxavir or oseltamivir within 48 h of disease onset, indicating the presence of fever. According to the prescription information [21,22], influenza-infected children received a single dose of oral baloxavir on day 1 (10 mg for those weighing 10–20 kg and 20 mg for those weighing 20–40 kg) or oral oseltamivir twice daily for 5 days (3 mg/kg for those aged 2 weeks to 12 months and 2 mg/kg for those aged 1–6 years on days 1–5). Depending on the individual’s symptoms, antipyretic, antitussive, expectorant, or antihistamine medications were administered as required. Fever resolution was defined as when multiple measurements taken several hours apart showed a temperature of <37.5 °C.

### 2.3. Study Methods and Statistical Analyses

Previous studies have suggested that influenza patients aged <18 years who were treated with baloxavir had a shorter fever duration than those treated with oseltamivir [14,15,17,18]. Fever resolution was selected as an index of clinical effectiveness. In this study, the primary endpoint was to compare the duration of fever between the oseltamivir- and baloxavir-treated groups and determine the clinical factors associated with days to fever resolution. Clinical factors were compared between the oseltamivir- and baloxavir-treated groups through univariate analyses using Welch’s *t*-test and Pearson’s chi-square test. The Kaplan–Meier curve of fever duration, which is an important index in this study, was constructed and compared between the groups using the log-rank test. Since some clinical factors were confounding factors, the independent clinical factors associated with fever duration were determined using multivariate logistic regression analysis. The analysis was binarized into 1 day of fever resolution and 2 or more days. Statistical significance was set at *p* < 0.05. All analyses were performed using SPSS software, V29.0.1 (IBM, Tokyo, Japan).

## 3. Results

### 3.1. Clinical Characteristics

**Figure 1:**
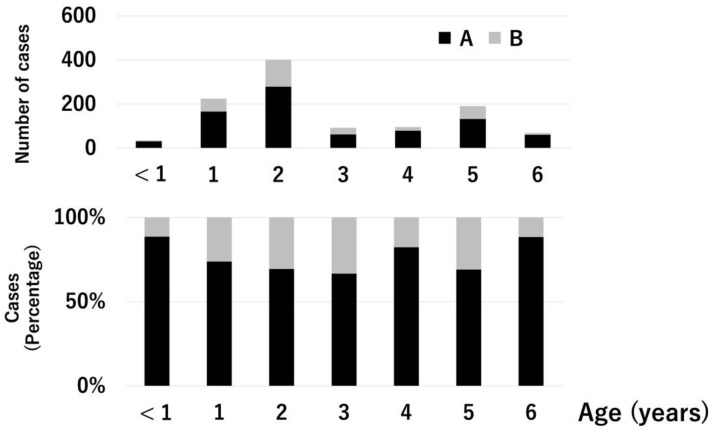
Prevalence of influenza types A and B based on age. The upper graph shows the number of cases. The lower graph shows the percentage. Black bar: influenza type A, gray bar: influenza type B.

**Figure 2:**
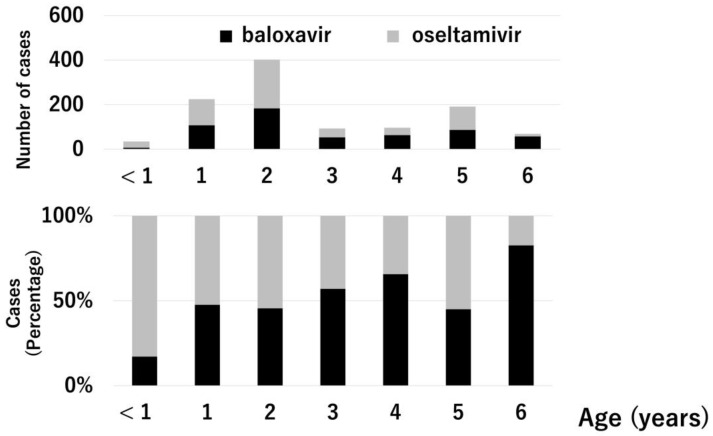
Comparison of treatment drugs based on age. The upper graph shows the number of cases. The lower graph shows the percentage. Black bar: baloxavir, gray bar: oseltamivir.

In this study, 1111 patients aged 0–6 years with influenza were analyzed (type A, 810 (72.9%); type B, 301 (27.1%); baloxavir-treated, 555 (50.0%); and oseltamivir-treated, 556 (50.0%)). Type A influenza is a primary epidemic in Japan. Patients in the 2015–2018 season predominantly received oseltamivir treatment, and those in the 2018–2019 season predominantly received baloxavir treatment because baloxavir treatment for influenza was approved for use in Japan in 2018. Figure 1 and Figure 2 show the influenza types and treatment drugs according to age. Type A was the most common type for all ages. Six (17.1%) of the 35 patients aged <1 year received baloxavir. Conversely, 57 (82.6%) of the 69 patients aged 6 years received baloxavir. Gastroenteritis symptoms such as vomiting and diarrhea were observed in 34 and 285 patients in the baloxavir- and oseltamivir-treated groups, respectively.

### 3.2. Comparison of Clinical Factors between the Baloxavir and Oseltamivir Treatment Groups

Except for sex and time from fever onset to drug administration, the other clinical factors were significantly different between the groups. In particular, the baloxavir group had significantly more patients with influenza type A, a shorter time from drug administration to the reduction in fever, fewer patients with upper respiratory tract symptoms 4–5 days after drug administration, and fewer patients who received influenza vaccination before the development of influenza than those in the oseltamivir group (Table 1).

### 3.3. Duration of Fever

**Figure 3:**
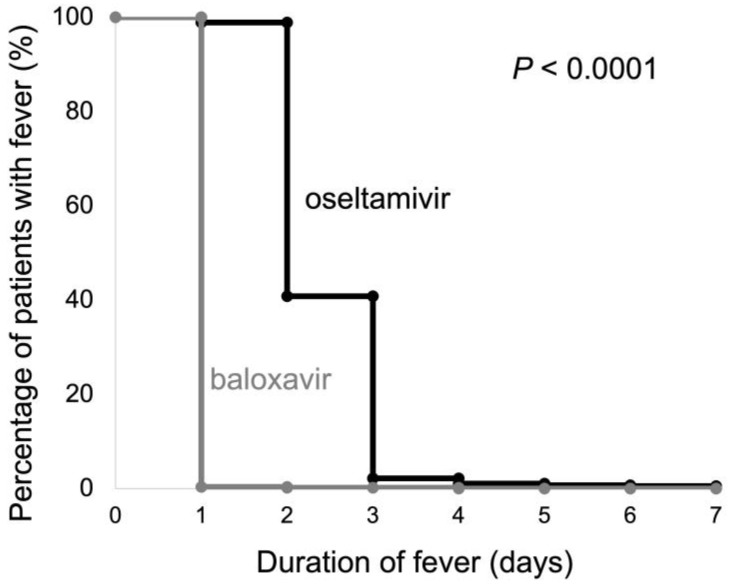
The Kaplan–Meier curves of the duration of fever in the baloxavir and oseltamivir treatment groups. Black line: oseltamivir, gray line: baloxavir.

Figure 3 shows the Kaplan–Meier curves for fever duration in the baloxavir and oseltamivir groups. The number of patients with a fever duration of one day was 553 (99.6%) in the baloxavir group and six (1.1%) in the oseltamivir group. The duration of fever was significantly shorter in the baroxavir group than in the oseltamivir group, according to the log-rank test (*p* < 0.001). In the multivariate logistic regression analysis, baloxavir use was the only independent clinical factor significantly associated with fever resolution (*p* < 0.001) (Table 2).

## 4. Discussion

This retrospective cohort study on early childhood influenza evaluated the clinical effectiveness of baloxavir treatment by comparing the duration of fever between the baloxavir and oseltamivir treatment groups. Most of the patients had resolved fever one day after baloxavir administration, which was one day earlier than the oseltamivir treatment. Even after adjusting for confounding clinical factors, sex, age, time from fever onset to drug administration, influenza type, and influenza vaccination, baloxavir use was an independent clinical factor associated with early fever resolution. Notably, the proportion of patients who received influenza vaccination was lower in the baloxavir treatment group than in the oseltamivir treatment group.

Although no previous studies have been conducted on influenza patients exclusively ≤6 years of age, some studies have examined the clinical effectiveness in pediatric patients with influenza treated with baloxavir. Baker et al. conducted a study in pediatric patients with influenza aged 1–12 years (miniSTONE-2) and showed similar clinical efficacy between the baloxavir and oseltamivir group [12]; the median durations of fever were 41.2 h (95% confidence interval [CI], 24.5–45.7) for baloxavir treatment and 46.8 h (95% CI, 30.0–53.5) for oseltamivir treatment. In the present study, the median time to fever resolution (one day after baloxavir administration) was shorter than the miniSTONE-2 result, although the fever resolution time was similar in patients treated with oseltamivir. The reason for this may have been the different doses of baloxavir or different numbers of patients between the two studies. The miniSTONE-2 also showed that median times to influenza symptom resolution were 138.1 h (95% CI, 116.6–163.2) for baloxavir treatment and 150.0 h (95% CI, 115.0–165.7) for oseltamivir treatment; indicating that influenza symptoms are continued for approximately 6 days. This is consistent with the results of the current study, as more than 80% of the patients continued to experience upper respiratory tract symptoms 4–5 days after drug administration.

In a Japanese clinical study, Kakuya et al. conducted a study of 235 pediatric and adolescent patients with influenza aged 3–18 years and compared the time to fever resolution between patients treated with baloxavir (*n* = 144) and those treated with oseltamivir (*n* = 91) [18]. The study found that the time to fever resolution with baloxavir treatment was significantly shorter than those with oseltamivir (overall influenza, baloxavir: median 22.0 h, oseltamivir: median 33.5 h, hazard ratio: 0.53 [95% CI, 0.38–0.73]). This finding is consistent with the results of the current study. Notably, their study was analyzed by type and subtype, with both types A and B having a significantly shorter time to fever resolution in the baloxavir-treated group than in the oseltamivir-treated group (type A, baloxavir: median 22.9 h, oseltamivir: median 28.5 h; type B, baloxavir: median 22.0 h, oseltamivir: median 55.0 h). This indicates that the time to fever resolution was more effective for type B than for type A. Furthermore, in the analysis by subtype, the hazard ratio was 0.55 (95% CI, 0.32–0.93) for A/H1N1 (pandemic 2009), with no significant difference for A/H3N2. This indicates that the efficacy of baloxavir treatment depends on the type and subtype of influenza. In the current study, baloxavir was predominantly used in patients with influenza type A because of the influenza A epidemic in 2018–2019. However, baloxavir treatment showed superior efficacy compared with oseltamivir treatment.

Influenza viruses with an amino acid substitution at position 38 of the RNA polymerase (polymerase acidic (PA) protein/I38X mutation) have been detected in patients treated with baloxavir [23], especially in young pediatric patients [12]. The mechanism of this emergence is thought to be that baloxavir has a strong virus-reducing effect, and wild-type viruses without the PA/I38X mutant disappear relatively quickly, resulting in the temporary appearance of viruses with only the PA/I38X mutant [24,25,26]. Because this virus has reduced susceptibility to baloxavir [23], there are concerns about the reduced clinical efficacy of baloxavir treatment and the transmission of the PA/I38X mutant virus among humans [25]. Regarding the clinical efficacy of baloxavir treatment in pediatric patients, it remains unclear whether the PA/I38X mutant virus prolongs symptom duration. Some clinical studies have reported a longer symptom duration in patients with the PA/I38X mutation than in patients without the PA/I38X mutation virus [12,19,27]; however, Saito et al. reported no significant differences in fever duration and symptom duration between patients with and without the PA/I38X mutation virus [28]. The PA/I38X mutant virus is not highly proliferative [24,29], and there are no reported epidemics of this virus so far. In the current study of early childhood patients, although no analyses of the mutant virus were performed, baloxavir treatment resulted in a short fever duration. Thus, in clinical practice, the PA/I38X mutation may not have a significant effect.

Our study had several limitations. As this was a retrospective cohort study, the number of cases studied varied by year and age, and the proportion of influenza types differed between the baloxavir and oseltamivir treatment groups. A potential selection bias might exist. In addition, the subtypes of influenza and the PA/I38X mutation virus were not analyzed in the current study, due to the clinical practice setting. Gastrointestinal symptoms could not be identified as a side-effect caused by influenza itself or anti-influenza drugs. Finally, since the current study was conducted in a Japanese primary care outpatient clinic, the time to the resolution of fever, which is the primary endpoint in this study, was analyzed in days and not hours after onset. Despite these limitations, the multivariate analysis clearly showed that baloxavir treatment was the only independent clinical factor associated with days to fever resolution.

## 5. Conclusions

This was a Japanese cohort study on early childhood influenza that compared the effectiveness of baloxavir and oseltamivir treatment. In early childhood influenza, baloxavir treatment resulted in a shorter fever duration than oseltamivir treatment. Further clinical studies including multi-center clinical trials are needed to validate this conclusion.

## Figures and Tables

**Table 1:** Comparison of clinical factors between baloxavir- and oseltamivir-treated groups.

Characteristics	Baloxavir *n* = 555	Oseltamivir *n* = 556	*p*-Value
Male	276 (50%)	281 (51%)	0.787
Age (years)	3.0 ± 0.07 (2.85–3.12)	2.5 ± 0.07 (2.39–2.66)	<0.001
Influenza type A B	550 (99%) 5 (1%)	260 (47%) 296 (53%)	<0.001
Time from fever onset to drug administration <24 h 24–48 h	474 (85%) 81 (15%)	474 (85%) 82 (15%)	0.942
Time from drug administration to resolution of fever (days)	1.0 ± 0.02 (0.97–1.05)	2.4 ± 0.02 (2.40–2.48)	<0.001
Patients with upper respiratory tract symptoms at 4–5 days after the drug administration	464 (84%)	550 (99%)	<0.001
Influenza vaccination Done Not done	227 (41%) 328 (59%)	359 (65%) 197 (35%)	<0.001

Data are shown as numbers (percent) or mean ± standard error (95% confidence interval). h, hour.

**Table 2:** Results of multivariate logistic regression analysis.

Clinical Factors	Odds Ratio (95% Confidence Interval)	*p*-Value
Male	0.576 (0.136–2.434)	0.453
Age	1.074 (0.689–1.675)	0.752
24–48 h from fever onset to drug administration	1.890 (0.212–16.805)	0.568
Influenza type A	0.551 (0.100–3.049)	0.495
Use of baloxavir	50,201 (5641–446,720)	<0.001
Influenza vaccination	1.593 (0.316–8.035)	0.573

h, hour.

## Data Availability

The data of this study are available from the corresponding author upon reasonable request.
